# The relationship between premenstrual syndrome and anger

**DOI:** 10.12669/pjms.35.2.232

**Published:** 2019

**Authors:** Havva Yesildere Saglam, Fatma Basar

**Affiliations:** 1 *Havva Yesildere Saglam, Department of Obstetrics and Gynecology Nursing, Faculty of Health Sciences, Eskisehir Osmangazi University, Eskisehir, Turkey*; 2 *Fatma Basar, Department of Obstetrics and Gynecology Nursing, Faculty of Health Sciences, Kutahya University of Health Sciences, Kutahya, Turkey*

**Keywords:** Anger, Premenstrual syndrome, Prevalence, Women

## Abstract

**Objective:**

Premenstrual Syndrome (PMS) is a significant disorder affecting the daily life of women of reproductive age. The aims of this study was to determine the PMS prevalence and the examination of the relationship between PMS and anger.

**Methods:**

This was a cross sectional study. The study was carried out with 720 women between the ages of 15-49 living in the province of Kutahya, Turkey. A Personal Information Form, the Premenstrual Syndrome Scale (PMSS) and the State-Trait Anger Scale (STAS) were used to collect data.

**Results:**

The PMS prevalence was 48.75%. There was a statistically significant difference between the groups in terms of constant anger, anger-in, anger-out and anger control subscales (p <.001). The average scores of women with PMS for constant anger anger-in and anger-out was significantly higher. The anger control scores were at a significantly lower level.

**Conclusion:**

Women with PMS had higher anger and lower anger control levels. It should be advisable to recommend anger control management and provide social support so these women can cope with the symptoms. The result of our study emphasizes the importance of careful assessment of anger in women with premenstrual symptoms.

## INTRODUCTION

PMS is a disorder characterized by physical, behavioral and emotional symptoms, which increases in severity during the luteal phase of the menstrual cycle.^1^ While the prevalence of PMS varies, 47.8% (32.6%-62.9%) was reported in a meta-analysis study.^2^ In women, physical symptoms such as breast tenderness, fluid retention leading to weight gain, fatigue, nausea, and constipation can occur in the premenstrual period. Also psychological symptoms such as the tendency to become angry, irritable, tense, anxious, and restless as well as behavioral symptoms like depression, nervousness, and crying are seen.^3^ Over 40 million women worldwide experience these symptoms.^4^ While approximately 90% of women have only mild premenstrual symptoms, approximately 20% have to cope with symptoms that severely disrupt their daily lives.^5^ Anger and irritability are one of the most severe and persistent symptoms of PMS that adversely affect women.^1^ It is reported that women frequently complain of anger and irritability in the premenstrual period.^6^ However, the relationship between anger and PMS has not been fully elucidated. There is not enough work on this subject. It is thought that hormonal changes in the menstrual cycle (fluctuations in estrogen and progesterone levels) affect the mood of women and trigger negative emotions such as anger and irritability. But no definitive evidence has yet been obtained on the relationship between PMS symptomatology and anger. There are few studies analyzing the relationship between PMS and anger. But, it is known that women who are able to control anger in daily life lose their anger management ability in the premenstrual period.

The aim of this study was to find out the prevalence of PMS, a common disorder affecting women throughout their reproductive years, and the relationship between PMS and anger. This study can contribute to the literature regarding the relationship between PMS and anger because there are limited studies available.

## Research Questions

- What is the prevalence of PMS in women aged 15-49?
- Is there a relationship between PMS and anger levels in women?

## METHODS

This cross-sectional study population consisted of 8230 women aged 15-49, who are registered in a family health center. The sample size of the study was calculated as 311 women with a 95% confidence interval and a 5% error margin, assuming a 30% incidence of PMS in the community. However, due to reliability of the study and the possibility of data loss, 720 women were initially contacted. The women included in the study were identified by the use of randomization tables. Study inclusion criteria were:

1. Age between 15-49 years old
2. Literate
3. Experiencing menstruation
4. Able to communicate openly
5. Agreeing to be in the study

Pregnant, lactating or menopausal women were not included in the study, as well as those who had undergone urogenital surgery or had a chronic disease. Furthermore, woman with psychiatric disturbances, difficulty establishing open and honest communication or who were outside the age parameters of 15-49 were not included in the study.

### Data Collection

Data were collected between October and December 2016 in a family health center in the central district of Kutahya, which has the largest female population in the area. A personal Information Form, the Premenstrual Syndrome Scale (PMSS) and the State–Trait Anger Scale (STAS) were used to collect data.

### Personal Information Form

The form includes questions about the women’s socio-demographic, fertility and menstruation characteristics.

### Premenstrual Syndrome Scale (PMSS)

PMSS was developed by Gencdogan to determine the severity of premenstrual symptoms.^7^ The scale is a 5-point Likert-type measure consisting of 44 items. The options are as follows: None (1 point), Very little (2 points), Sometimes (3 points), Often (4 points), Continuous (5 points). The lowest PMSS score is 44 and the highest score is 220. More than 50% of the total PMSS scores were classified as PMS positive. Higher PMSS scores indicate greater symptom severity during PMS. The Cronbach’s alpha coefficient of the scale is 0.75.^7^ In this study, the Cronbach’s alpha coefficient of the scale was 0.95.

### The State–Trait Anger Scale (STAS)

The scale was developed by Spielberger to determine the manner of anger expression. It was translated and adapted into Turkish by Ozer. 8 The scale is a Likert-type measure consisting of 34 items. The options are evaluated as follows: Never (1 point), A little (2 points), Quite (3 points) and Very (4 points). The scale consists of Constant Anger, Anger-in, Anger-out and Anger Control subscales. High scores from constant anger show a high level of anger. High scores from the anger-in subscale indicate suppressed anger and high scores from the anger-out subscale indicate anger control issues. A high score from the anger control subscale indicates that the anger can be controlled.

The Cronbach alpha coefficients for the scale were 0.79 for the “constant anger” subscale, 0.78 for the “anger-out subscale”, 0.62 for the “anger-in subscale” and 0.84 for the “anger control subscale”.^8^ The Cronbach alpha coefficients for the scale of this study were 0.84 for the “constant anger” subscale, 0.79 for the “anger-out subscale”, 0.66 for the “anger-in subscale” and 0.82 for the “anger control subscale”

### Statistical Analysis

The data were analyzed using SPSS Statistic Version 22.0 (SPSS, Inc., Chicago, IL, USA). Chi square (X^2^) analysis was performed to examine the relationship between PMS status and categorical variables (socio-demographic characteristics). To understand the difference between the two groups, t-test (t) was used in independent groups. Pearson-Correlation test was applied in the analysis of the relationship between PMS scores and constant anger and anger expression style scores. The results were evaluated at a significance level of p<0.05 and p<0.001.

### Ethical Considerations

The purpose of the study was explained to each woman and their consent obtained. The ethical approval was obtained from the Eskisehir Osmangazi University Ethics Committee for Non-Interventional Clinical Investigations (80558721/G-252) dated 15.08.2016. The research permit (66581584/730.08.03) dated 05.09.2016 was issued by the Public Health Directorate of Kutahya. The authors have no ethical conflicts to disclose.

## RESULTS

The prevalence of PMS was 48.75%. There was no statistically significant difference between groups in terms of socio-demographic characteristics (except marital status) (p>0.05). 34.2% of the women with PMS were in the 15-24 age groups, 38.5% were university graduates, 60.7% were married and 73.2% were not working. Of the non-PMS women, 37.4% were in the 25-34 age group, 38.2% were university graduates, 68.3% were married, and 73.7% were not working (Table-I).

**Table-I:** Socio-demographic characteristics of the women.

	Without PMS (51.25%)		With PMS (48.75%)			

Variable	n=369	%	n =351	%	X^2^	p
***Age***						
Age 15-24	100	27.1	120	34.2	4.373	0.112
Age 25-34	138	37.4	115	32.8		
Age 35-49	131	35.5	116	33.0		
***Educational Status***						
Primary school	111	30.1	103	29.3	0.049	0.976
High school	117	31.7	113	32.2		
University and above	141	38.2	135	38.5		
***Working status***						
Yes	97	26.3	94	26.8	0.022	0.881
No	272	73.7	257	73.2		
***Income Status***						
Income more than expenses	54	14.6	38	10.8	4,060	0.131
Income matches expenses	255	69.1	240	68.4		
Income less than expenses	60	16.3	73	20.8		
***Marital Status***						
Single	117	31.7	138	39.3	4.553	0.033*
Married	252	68.3	213	60.7		

* p<0.05

The mean age of first menarche of women with PMS is 13.30 ± 1.49, the menstrual cycle interval was 26.75 ± 4.38 days and the duration in days of menstrual bleeding is 6.10 ± 1.50. Women without PMS have a mean age at first menarche of 13.40 ± 1.47, a menstrual cycle interval of 26.32 ± 3.99 days and duration in days of menstrual bleeding of 6.15 ± 1.62. 71.5% of women with PMS have a regular menstrual cycle, while 86% have dysmenorrhea and 81.3% of Non-PMS Women has regular menstrual cycle with 67.2% dysmenorrhea. There was no statistically significant difference between the groups in terms of menarche age, menstrual cycle interval and menstrual bleeding durations (p>0.05). There is a statistically significant difference between the groups with and without PMS in terms of regular menstrual cycle, dysmenorrhea and the number of pregnancies (p<0.05). An irregular menstrual cycle, dysmenorrhea and no pregnancies were associated with PMS (Table-II).

**Table-II:** Menstruation and fertility characteristics of the women.

Variable	Without PMS		With PMS		t	p

	Error! Reference source not found.±SD		Error! Reference source not found.±SD			
Age of menarche	13.40±1.47		13.30±1.49		0.896	0.370
Menstrual cycle interval	26.32±3.99		26.75±4.38		-1.367	0.172
Menstrual bleeding duration in days	6.15±1.62		6.10±1.50		0.396	0.692

*Variable*	*Without PMS*		*With PMS*		*X^2^*	*p*

	*n=369*	*%*	*n =351*	*%*		

***Menstrual cycle regularity***						
Regular	300	813	251	715	9.600	0.002*
Irregular	69	18.7	100	28.5		
***Dysmenorrhea***						
Yes	248	67.2	302	86.0	35.368	<0.001**
No	121	32.8	49	14.0		
***Number of pregnancies***						
N/A	126	34.1	161	45.8	11.797	0.008*
1	77	20.9	54	15.4		
2	97	26.3	87	24.8		
3 or more	69	18.7	49	14.0		

* p<0.05

The mean scores of women with PMS were 21.87 ± 5.81 for constant anger, 16.93 ± 3.97 for anger-in, 16.03 ± 4.53 for anger-out and 20.22 ± 4.39 for anger control. The mean score of Non-PMS Women were 17.98 ± 5.14 for constant anger, 14.75 ± 3.76 for anger-in, 13.98 ± 3.74 for anger-out and 21.40 ± 4.79 for anger control. There was a statistically significant difference between the groups in terms of constant anger, anger-in, anger-out and anger control subscales (p<0.001). The average scores of women with PMS for constant anger anger-in and anger-out was significantly higher. In addition, the anger control scores were at a significantly lower level (Table-III).

**Table-III:** The relationship between PMS and the STAS subscales score averages.

Trait Anger	Anger-in	Anger-out	Anger Control

	Error! Reference source not found.±SD	Error! Reference source not found.±SD	Error! Reference source not found.±SD	Error! Reference source not found.±SD
Without PMS	17.98±5.14	14.75±3.76	13.98±3.74	21.40±4.79
With PMS	21.87±5.81	16.93±3.97	16.03±4.53	20.22±4.39
	p<0.001	p<0.001	p<0.001	p= 0.001
	t=-9.495	t=-7.565	t=-6.573	t=3.451

There was a statistically significant positive moderate correlation between the total score from PMSS and the scores obtained from the subscales for constant anger, anger-in and anger-out (p<0.001). There was a statistically significant negative weak correlation between the total score from PMS and the scores from the subscales for anger control (p=0.001). There was a significant relationship between the PMS scores of the women and the scores for constant anger, anger-in, anger-out, and anger control (Table-IV).

**Table-IV:** The relationship between women’s PMSS scores and STAS subscale scores.

		State–Trait Anger Scale Score			

		Trait Anger	Anger-in	Anger-out	Anger Control
*PMSS Score*	r	0.479	0.356	0.348	-0.181
	p	p<0.001	p<0.001	p<0.001	p<0.001

## DISCUSSION

The study results demonstrated that approximately half of the women suffered PMS (48.75%). Rezaa H et al. found the prevalence of PMS was 52.9%.^9^ In a meta-analysis study, the prevalence of PMS was reported to be 47.8%.^4^ In 2 different research studies carried out on women in the reproductive age group between 15-49 years in Turkey, the prevalence of PMS was seen to be 40% and 90% respectively.^10^,^11^

In this study, there was a statistically significant difference between the presence of PMS and anger (p<0.05). Women with PMS had higher anger and lower anger control levels. In literature it was reported that anger and irritability are one of the most severe and persistent symptoms of PMS.^6^,^12^,^13^ But there are few studies analyzing the relationship between PMS and anger.^12^-^14^ Ducasse D et al.^5^ found an impulsive-aggressive pattern of personality in women with PMS independently from the time of the menstrual cycle. Interestingly, trait anger remained associated with PMS independently of every other personality traits. In a study of Bostanci, analyzing the anger and anxiety levels of healthy and PMS-women, PMS-women were found to have consistently higher scores in anger, anger-in, anger-out and lower scores in terms of anger control.^14^ Ozturk Can H et al. found in a study with teachers that the anger levels of women with PMS are high but there was no significant relationship between PMS and anger control scores.^15^ Reihane et al. found the mean scores of psychiatric symptoms (Depression, Anxiety, Aggression, Interpersonal sensitivity) in the PMS group were significantly higher than the healthy group. There was a significant difference in mean score of depression, anxiety, aggression and interpersonal sensitivity between the 3rd and the 13th day of the cycle.^16^

The correlation between women’s PMS scores and anger scores were significant in this study. Similarly, the study of Soyda Akyol E et al. showed that PMS scores and anger scores were associated with premenstrual dysphoric disorder.^17^ Bowen R et al. found that women with PMS had more nervousness and depression. These differences were present regardless of the late luteal phase.^18^ Van der Ploeg investigated premenstrual affect changes with 844 women and found that women with PMS had higher anxiety, anger and depression scores.^19^ In the Hartlage and Arduino study, premenstrual disorders are also associated with anger.^12^ Similar to the literature, our results support a relationship between PMS and anger. Except these studies, there is no study examining the relationship between PMS and anger in the literature.

## CONCLUSION

PMS are common disorders among women. As anger is among the most common symptoms in the premenstrual period, the alleviation of anger levels could increase the quality of life for women. In this context, it may be advisable to recommend anger control management and provide social support so these women can cope with the symptoms. The result of our study emphasizes the importance of careful assessment of anger in women with premenstrual symptoms. More advanced studies are needed to provide definitive evidence about the relationship between PMS and anger and to eliminate the gaps in the literature.

### Author`s Contribution

**HYS** conceived, designed, did data collection, manuscript writing and final approval of manuscript.

**FB** conceived, designed, did review, editing of manuscript and final approval of manuscript.
